# A Conjugate of Pentamethine Cyanine and ^18^F as a Positron Emission Tomography/Near-Infrared Fluorescence Probe for Multimodality Tumor Imaging

**DOI:** 10.3390/ijms18061214

**Published:** 2017-06-07

**Authors:** Fei-Fei An, Harikrishna Kommidi, Nandi Chen, Richard Ting

**Affiliations:** 1 Molecular Imaging Innovations Institute (MI3), Department of Radiology, Weill Cornell Medical College, 413 East 69th Street, New York, NY 10065, USA; fea2008@med.cornell.edu (F.-F.A.); hak2027@med.cornell.edu (H.K.); nac2038@med.cornell.edu (N.C.); 2 State Key Laboratory of Chemo/Biosensing and Chemometrics, College of Chemistry and Chemical Engineering, Key Laboratory for Bio-Nanotechnology and Molecular Engineering of Hunan Province, Hunan University, Changsha 410082, China

**Keywords:** PET, near-infrared, multimodality, bio-imaging, cyanine dyes, ^18^F

## Abstract

The novel synthesis of a dual-modality, pentamethine cyanine (Cy5) fluorescent, ^18^F positron emission tomography (PET) imaging probe is reported. The probe shows a large extinction coefficient and large quantum yield in the biologically transparent, near-infrared window (650–900 nm) for in vivo fluorescent imaging. This fluorophore bears the isotope, ^18^F, giving a ^18^F-PET/near-infrared fluorescent (NIRF), bi-modal imaging probe, that combines the long-term stability of NIRF and the unlimited penetration depth of PET imaging. The bi-modal probe is labeled with ^18^F in a quick, one-step reaction, which is important in working with the rapid decay of ^18^F. The bi-modal probe bears a free carboxyl group, highlighting a PET/NIRF synthon that can be conjugated onto many advanced biomolecules for biomarker-specific in vivo dual-modal PET/NIR tumor imaging, confocal histology, and utility in multi-fluorophore, fluorescence-guided surgery. Its potential in vivo biocompatibility is explored in a quick proof-of-principal in vivo study. The dye is delivered to A549 xenograft flank-tumors to generate PET and NIRF signals at the tumor site. The tumor distribution is confirmed in ex vivo gamma counting and imaging. Pentamethine cyanine (Cy5) has the ability to preferentially accumulate in tumor xenografts. We substitute the PET/NIRF probe for Cy5, and explore this phenomenon.

## 1. Introduction

Cyanine dyes are widely used as fluorescent probes for bio-imaging because of their high extinction coefficients, biocompatibilities, and high sensitivities [1,2]. The clinical utility and biocompatibility of Indocyanine Green (ICG) has inspired many different biomedical applications of cyanine dyes [3,4,5,6]. Recent reports have investigated cyanine dye accumulation in tumors intrinsically, allowing for fluorescence-based, tumor-targeted imaging [7,8,9,10]. Unfortunately, fluorescence imaging through deep tissue is limited by complications associated with exciting and emitted photon scattering and nonspecific photon absorption of biological tissue [11,12,13]. This drawback can be addressed by combining fluorophores with radioemitters so that dual-/multi-modality imaging could be used for comprehensive, quantitative, deep-tissue imaging [14,15,16,17].

The combination of a positron emission tomography (PET) emitter and a near-infrared (650–900 nm) fluorescence (NIRF) probe is an attractive amalgamation [15]. A dual-modality PET/NIRF synthon exploits the non-invasive and unlimited penetration of PET imaging and the high local spatial resolution of NIR fluorescence imaging to guide intraoperative resection or post-surgery pathological examination to ultimately improve clinical outcome [18,19,20,21].

In the past, the isotope ^64^Cu has been used to label cyanine dyes to give bi-modal imaging probes that combine the advantages of both fluorescence and positron emission tomography (PET) [22]. ^64^Cu is not a widely-used isotope in the clinic. This may be due to ^64^Cu’s relatively long half-life (*t*_1/2_ = 12.7 h), low β^+^ decay purity (18%), and the mean energy of the emitted positron [15]. Alternatively, ^18^F is a very popular clinical isotope for PET imaging, because of its relatively shorter half-life (*t*_1/2_ = 108 min) and high β^+^ purity (97%) of decay. These properties translate into fewer biological side effects and less environmental contamination.

Unfortunately, ^18^F-labeled agents have been traditionally difficult to synthesize, due to the harsh or time-consuming chemistry of fluoride-carbon bond formation [23,24]. The extended conjugation present in fluorophores makes cyanine dyes especially difficult to directly label with ^18^F, as they are sensitive to chemical decomposition. Yet, the popularity of indocyanine dyes in the clinic make PET/fluorescent indocyanines attractive multimodality targets. Of the available cyanine dyes, pentamethine cyanines (Cy5) have the ideal NIRF optical properties (*λ*_max_ = 661 nm, *ϵ*_661nm_ = 249,000, *λ*_ex_ = 660 nm, *λ*_em_ = 679 nm, *ϕ*_H2O_ = 0.19), specifically, absorbance in the NIRF window, large extinction coefficients, and a large quantum yield.

**Figure 1:**
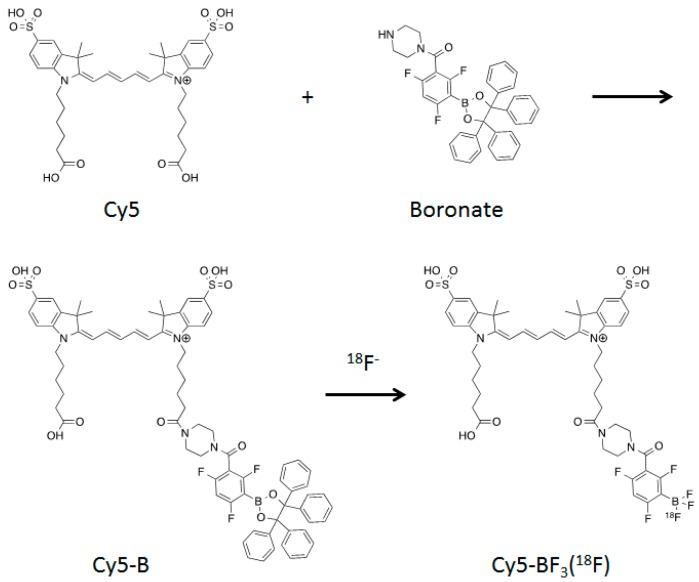
Synthetic scheme for a Cy5-BF_3_ (^18^F). ^18^F-labeled conjugate for PET/NIRF dual-modal imaging.

Herein, we labeled Cy5 dye with ^18^F to generate a superior PET/NIRF dye for bi-modal imaging. We tested its utility on xenografted A549 flank-tumors for tumor-targeted imaging and image-guided surgery. A Cy5-boronate conjugate is first synthesized and then labeled with ^18^F in a rapid one-step reaction (Figure 1). The labeled conjugate can be incubated with A549 cells in vitro without observed cytotoxicity. The labeled conjugate is intravenously injected into tumor-bearing mice and results in non-invasive tumor PET imaging and confirmation of tumor targeting by ex vivo fluorescence. These results demonstrate the immediate utility of the ^18^F-labeled Cy5 conjugate as a PET/NIR fluorescence dual-modality probe for tumor imaging, imaging guided surgery, post-surgery pathological examination, and potential application in future advanced biomolecular conjugates.

## 2. Results and Discussions

### 2.1. Synthesis and Fluoride Reaction

Cy5 is conjugated to boronate by carbodiimide-coupling to give Cy5-B. This reagent could be labeled with ^18^F in a one-step reaction with 100 mCi ^18^F^−^ at 50 °C (Figure 1). A solution of non-radioactive, ^19^F-hydrogen fluoride carrier drives the reaction to completion and simultaneously buffers the reaction at pH 3 [25,26,27,28]. The conversion is confirmed by LC-MS. The mass of Cy5-B ([M]^+^) is 1343.7 (calculated [M]^+^ = 1343.5 *m*/*z*), which is the mass of Cy5 ([M]^+^ = 743.3) plus boronate ([M]^+^ = 619.3), minus a water molecule that is removed during coupling (Figures S1–S4).

### 2.2. Radiolabeling

**Figure 2:**
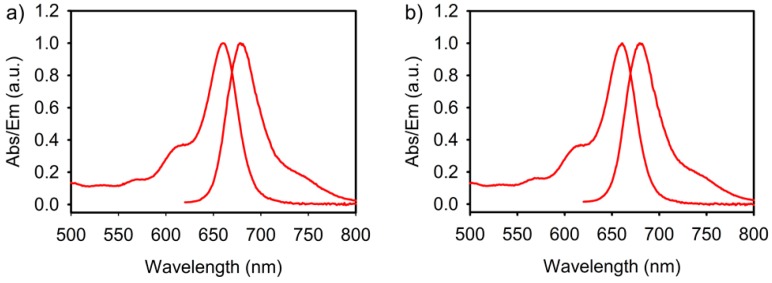
(**a**) The absorption and emission spectra of the Cy5-B in DMSO; (**b**) The absorption and emission spectra of the Cy5-BF_3_ in DMSO. (Excitation (Ex) = 600 nm, Emission (Em) = 680 nm).

^18^F labeling takes ~1 h without optimization. Rapid synthesis is critical in working with for ^18^F, which decays rapidly (half-life = 110 min). A 1 h incubation at 50 °C is more than sufficient for complete Cy5-B conversion. This time is unoptimized. In the future, this synthesis can be made to proceed more rapidly using higher temperatures and/or a microwave reactor. Cy5-BF_3_ (^19^F) is confirmed by LC-MS (LC analysis following radiodecay; calculated for [M]^−^ = (C_48_H_53_BF_6_N_4_O_10_S_2_)^−^ = 1034.3 *m*/*z*, [M]^2−^ = 517.2 *m*/*z* (double charged), observed [M]^2−^ = 517.6 *m*/*z*). The Cy5-BF_3_ (^18^F) shows an absorption and fluorescence emission spectrum similar to Cy5-B (absorption peak at ~660 nm, and emission peak at ~680 nm) (Figure 2a). The 650 nm wavelength can be used in monitoring the conjugate during purification with C18 Reversed Phase LC Column. The emission peak at ~680 nm is located in the near-infrared (NIR) transparent biological window (650–900 nm), and optical range is preferred for clinical translation because of its deeper penetration through overlying biological tissue [29,30,31,32,33]. The absorption and emission spectra of Cy5-B and Cy5-BF_3_ (^18^F) are nearly identical. Boronate or trifluoroborate does not change the fluorescent properties of Cy5 (Figure 2 and Figure S5).

**Figure 3:**
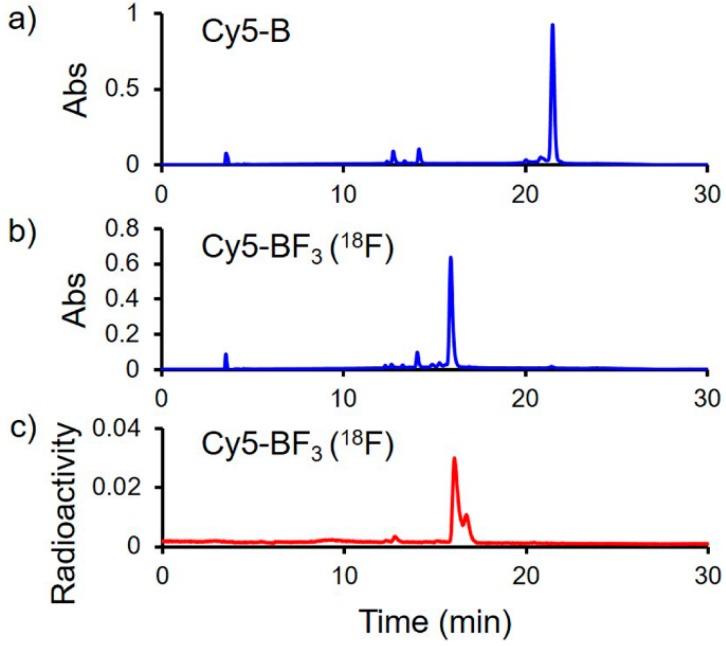
(**a**) The HPLC shift of the Cy5-B in the absorption channel of 650 nm wavelength; (**b**) The HPLC shift of the Cy5-BF_3_ (^18^F) in the absorption channel of 650 nm wavelength; (**c**) The HPLC shift of the Cy5-BF_3_ (^18^F) in the radioactivity channel of Cy5-BF_3_ (^18^F). C18 Reversed Phase LC Column (Waters, SunFire C18 Column, 100 Å, 3.5 µm, 3 mm × 100 mm, water/acetonitrile: 90/10 to 10/90, 20 min, ultrapure water and HPLC acetonitrile (Sigma, St. Louis, MO, USA)).

Benzopinacol on Cy5-B is highly hydrophobic, giving rise to a delayed elution time of 21.5 min in a C18 Reversed Phase HPLC Column (Figure 3a). The purity was also confirmed with HPLC in the channel of Cy5 absorption, as is shown in Figure S6. In the presence of hydrogen fluoride (HF), the benzopinacol is cleaved from the conjugate and the fluoride atoms bind to the boron atom by covalent bonds. The replacement of benzopinacol with fluoride during ^18^F labeling results in a species that is much more hydrophilic. Cy5-BF_3_ (^18^F) elutes at 15.9 min, allowing the easy separation of a very hydrophilic derivative (Figure 3b) from the hydrophobic starting material. A radioactive elution peak at 15.9 min corroborating Cy5-BF_3_ (^18^F) absorbance is confirmed with a radiodetector that is attached to the HPLC (Figure 3d). A further confirmation of the purity is conducted with HPLC in the channel of Cy5 absorption (Figure S7). The proton integration ratio between the Cy5 and the cleaved boronate confirm the purities very well (Figure S8). The correlation between the retention times absorption and the radioactivity channels of Cy5-BF_3_ (^18^F) indicates a successful labeling of ^18^F onto the Cy5.

### 2.3. MTT Assay

**Figure 4:**
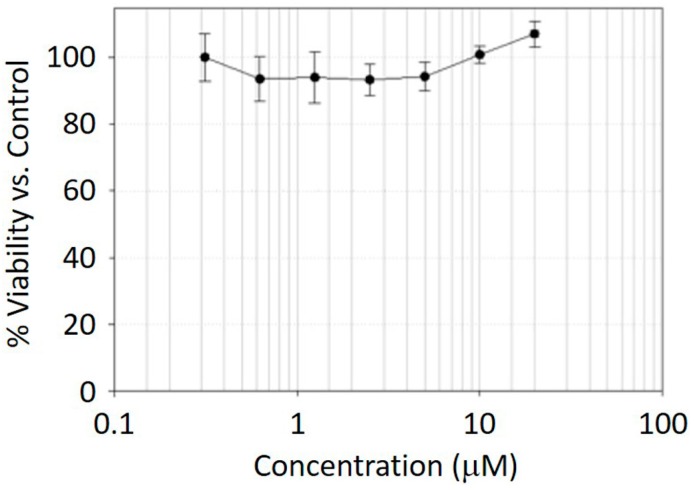
MTT cell viability tests with different concentrations of Cy5-BF_3_ (^19^F) after 24 hour of incubation with A549 cells.

A549 lung cancer cells were placed in a 96-well plate and incubated with Cy5-BF_3_ (^19^F) at different concentrations for 24 h. Standard cell metabolic activity (3-(4,5-dimethylthiazol-2-yl)-2,5-diphenyltetrazolium bromide, MTT) assays show that the PET/NIRF probes are not cytotoxic even at concentrations as high as 20 μM (Figure 4). This result suggests that Cy5-BF_3_ (^18^F) may be safe for in vivo applications.

### 2.4. Hemolysis Assay

**Figure 5:**
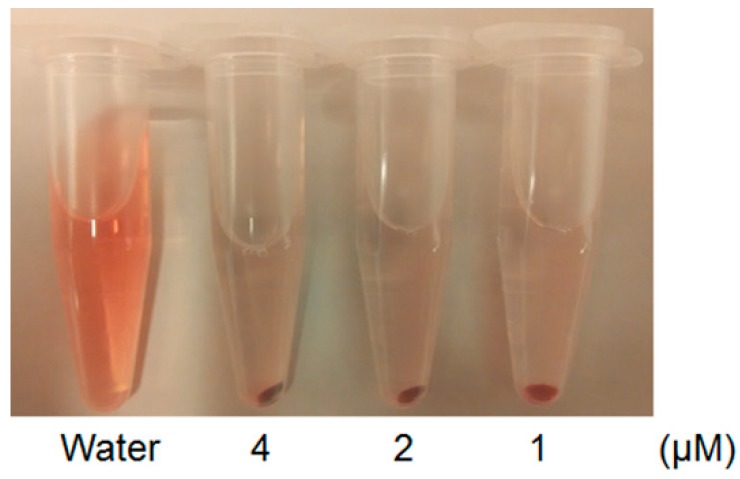
Hemolysis assay of Cy5-BF_3_ (^19^F) after 4 h of incubation with red blood cells (RBCs). Complete RBC hemolysis is observed in deionized water (control, left, shows lack of pelleting and red colored supernatant). Hemolysis is not observed with 4, 2, or 1 µM Cy5-BF_3_ in isotonic phosphate-buffered saline (pH = 7.4, PBS).

The potential risk of hemolysis induced by the probe was evaluated. Cy5-BF_3_ (^19^F) at different concentrations was incubated with red blood cells (RBCs) for 4 h. The result of the hemolysis assay shows that red blood cells mostly maintain their intactness after 4 h of incubation with Cy5-BF_3_ (^19^F) at a concentration as high as 4 μM. This concentration is larger than that used in cell imaging (Figure 5). As a control, red blood cells incubated in deionized water show total lysis due to the osmotic pressure placed on the cell membrane. Complete lysis is observed. The solution appears red and no pellet is produced upon centrifugation. RBCs incubated with the probe do not color the supernatant, but produce an intact pellet under centrifugal force, confirming the biocompatibility of the synthesized probe.

### 2.5. In Vitro Cell Imaging and Fluorescence Activated Cell Sorting (FACS) Quantification

**Figure 6:**
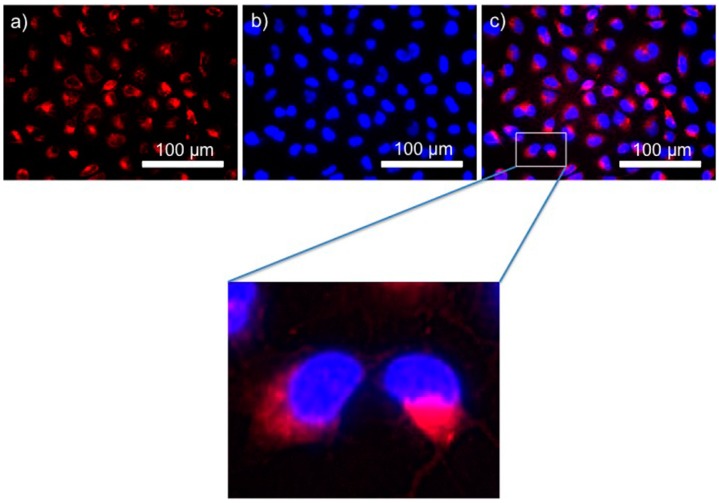
(**a**) Fluorescence imaging of A549 cells after incubating with Cy5-BF_3_ (^19^F) (2 μM, 1 h, red); (**b**) Fluorescence imaging of A549 cells after incubating with Hoechest 33258 (1 μg/mL, 1 min, blue); (**c**) The overlay of (**a**,**b**).

Cy5-BF_3_ (^19^F) can stain cells. A549 cells were incubated with 1 μM Cy5-BF_3_ (^19^F) in a 96-well plate. One hour later, the cells were incubated with 1 μg/mL Hoechest 33258 for 1 min. After being washed with 1× PBS, the A549 cells were imaged with fluorescence microscopy at the Cy5 channel. Cy5-BF_3_ (^19^F) fluorescence shows around the nuclei of cells (Figure 6b–d), indicating that Cy5-BF_3_ could be taken up by cancer cells and could be used for cell imaging.

**Figure 7:**
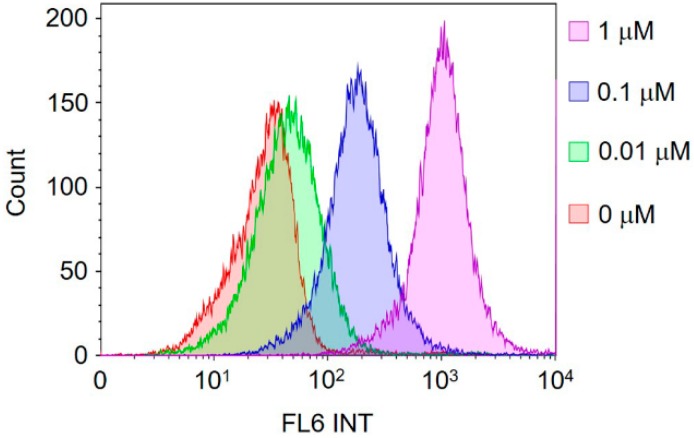
FACS quantification of A549 cells fluorescence intensities after 1 h of incubation with Cy5-BF_3_ (^19^F) at different concentrations.

To confirm the fluorescent labeling ability of Cy5-BF_3_ (^19^F), different concentrations of Cy5-BF_3_ (^19^F) were incubated with A549 cells. After 1 h of incubation, the fluorescent intensity of A549 cells was quantified with FACS at the channel for Cy5. It is found that the fluorescent intensities of A549 cells are highly dependent on the Cy5-BF_3_ (^19^F) concentrations used to label the cells. At higher concentrations, Cy5-BF_3_ (^19^F) shows a larger fluorescence shift (Figure 7) by FACS. The fluorescence intensity of A549 cells incubated with 0.01 μM Cy5-BF_3_ (^19^F) is less in comparison with untreated A549 cells. The result indicates that more Cy5-BF_3_ (^18^F) is preferred for in vivo tumor imaging, especially for fluorescence imaging. In translation, it may be necessary to supplement Cy5-BF_3_ (^18^F) with non-radioactive Cy5-BF_3_ (^19^F) to achieve an optimal in vivo optical signal. Note that this would lead to the dilution of Cy5-BF_3_ (^18^F) specific activity.

### 2.6. In Vivo PET Imaging and In Vitro Fluorescence Imaging

**Figure 8:**
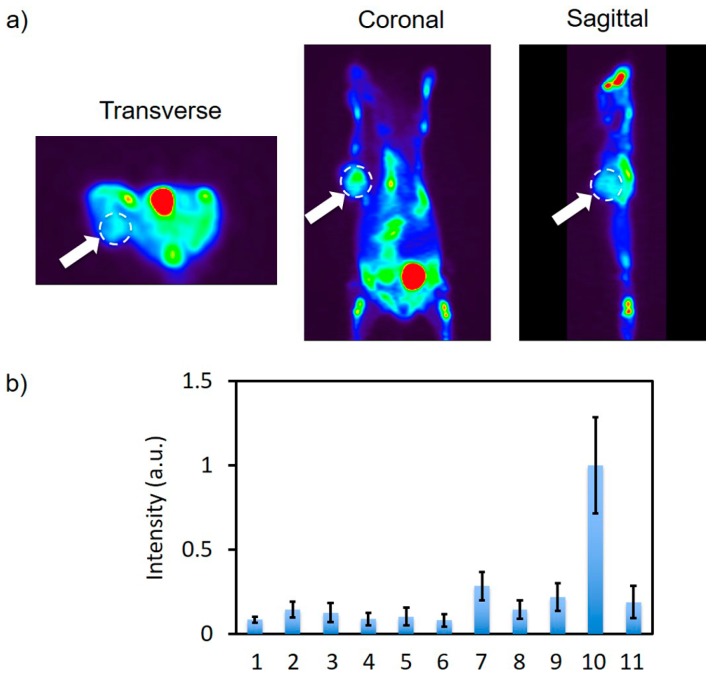
(**a**) Tomographic PET imaging of a flank A549 tumor 6 h after intravenously injecting the Cy5-BF_3_ (^18^F). The white dashed circles indicate the tumor; the brightest spot (red) is the bladder, indicating renal clearance of the probe (**b**) the quantitative biodistribution by gamma counter (Wallac Wizard 3.0, %ID normalized to tumor) 6 h after intravenously injecting Cy5-BF_3_ (^18^F). The data are normalized on the tumor. 1—Heart, 2—Liver, 3—Spleen, 4—Lung, 5—Kidney, 6—Stomach, 7—Intestine, 8—Bone, 9—Muscle, 10—Tumor, 11—Brain.

A ~60 μCi quantity of Cy5-BF_3_ (^18^F) in 1× PBS buffer (pH 7.4) was intravenously injected into the A549 flank of tumor-bearing Bal b/c mice. Six hours later, mice were imaged on an Inveon PET/CT (Siemens, Princeton, NJ, USA) scanner. We attempt to observe the PET signal at the position of the flank A549 tumor (Figure 8a). To confirm the tumor-specific ^18^F signal, mice were sacrificed, organs were collected, and scanned with gamma counter. The results show some distribution to the tumor in relation to other organs (Figure 8b).

**Figure 9:**
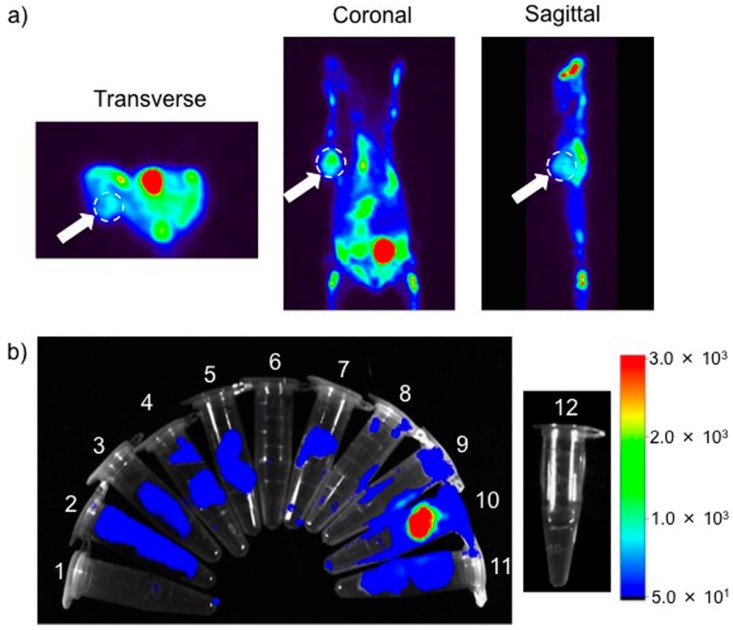
(**a**) Overlay of ex vivo fluorescence imaging and bright field image of the collected organs; (**b**) The semi-quantitative biodistribution by fluorescence imaging 6 h after intravenously injecting the Cy5-BF_3_ (18F). 1—Heart, 2—Liver, 3—Spleen, 4—Lung, 5—Kidney, 6—Stomach, 7—Intestine, 8—Bone, 9—Muscle, 10—Tumor, 11—Brain, 12—1× PBS.

The collected organs are also imaged with Bruker Xtreme Imaging System. A strong, confirmative fluorescence signal at the tumor indicates some accumulation of Cy5-BF_3_ (^18^F) at the tumor (Figure 9a). The semi-quantitative analysis of the organs showed distribution to the tumor (Figure 9b). The Cy5-BF_3_ (^18^F) bimodal probe could be used for non-invasive PET imaging in tumor diagnosis, fluorescence-guided tumor resection, and fluorescence confirmation of a successful surgery.

## 3. Materials and Methods

### 3.1. Cy5-B Synthesis

Cy5 dicarboxylate (22.3 mg, 0.03 mmol) and boronate (18.6 mg, 0.03 mmol) were synthesized according to procedures that have been reported previously [34,35,36,37,38]. All the other solvents and reagents were purchased from Sigma-Aldrich (St. Louis, MO, USA). Reagents were added to a round-bottom flask in the following order: Cy5 dicarboxylate (22.3 mg, 0.03 mmol) [34], boronate (18.6 mg, 0.03 mmol) [35,36,37,38], *N*-(3-Dimethylaminopropyl)-*N*′-ethylcarbodiimide hydrochloride (EDC, 8.9 mg, 0.045 mmol, Sigma), 1-Hydroxybenzotriazole hydrate (HOBt, 6.9 mg, 0.045 mmol, Sigma), *N*,*N*-Dimethylformamide (DMF, 2 mL, Sigma, HPLC), and pyridine (32 μL, Sigma, St. Louis, MO, USA) were stirred with a magnetic bar overnight and then purified with C18 Reversed Phase LC Column (00G-4253-P0-AX, Phenomenex (Torrance, CA, USA), Luna^®^ 10 µm C18(2) 100 Å, LC Column 250 × 21.2 mm, water/acetonitrile: 90/10 to 10/90, 20 min, ultrapure water and HPLC acetonitrile (Sigma, St. Louis, MO, USA)). The product fractions eluting between 22.5 min and 25 min were collected, frozen, lyophilized to a green powder, and stored at −80 °C. Yield: 4.3 mg (10.7%). The product was confirmed by LC-MS (Waters Acquity TQD LC/MS/MS System, Milford, MA, USA).

### 3.2. ^18^F Radiolabeling

16 nmol of Cy5-B was dissolved in 20 μL of tetrahydrofuran (THF). A 20-μL HF solution (0.6 M in water) containing 100 mCi of ^18^F^−^ ion was added. After 1 h of incubation at 50 °C, the mixed solution was quenched with EtOH/NH_4_OH (95/5, *v*/*v*, Sigma, St. Louis, MO, USA) and purified with over a plug of 200 mesh silica gel according to our previous protocol for purifying the boronate-based ^18^F labeling [22,23,24,34]. The mixed solution was loaded into a 10-mL volume syringe that was filled with 5 mL 200 mesh silica gel. Cotton was filled at the outlet before filling with silica gel to avoid silica gel leakage. The radiotracer was collected with EtOH/NH_4_OH (95/5, *v*/*v*, Sigma) as the eluent. The excessive ^18^F was retained in the silica gel. The collected radioactive, fluorescent, blue fraction was dried for 15 min with nitrogen, and then re-dissolved in 1× PBS buffer (pH 7.4) for intravenous (i.v.) tail vein injection.

### 3.3. MTT Assay

The biocompatibility of this conjugate was determined in a standard 3-(4,5-dimethylthiazol-2-yl)-2,5-diphenyltetrazolium bromide (MTT) assay. A549 cells were pre-seeded in 96-well plates (~5000 cells/well) and then cultured with RPMI 1640 (Gibco, Gaithersburg, MD, USA) supplemented with 10% Fetal Bovine Serum (FBS, Gibco, Gaithersburg, MD, USA), penicillin (100 μg·mL^−1^), and streptomycin (100 μg·mL^−1^; Gibco) in 5% CO_2_ at 37 °C in a humidified incubator for 24 h. The conjugate at different concentrations was added and cultured in 5% CO_2_ at 37 °C in a humidified incubator for a further 24 h. MTT at a final concentration of 1 mg/mL was incubated with cells for 4 h, and then cells were washed with 1× PBS buffer (pH 7.4) three times. After removing all of the solution, 100 μL DMSO was added to each well of the 96-well plate and read with a Tecan Infinite M1000 Pro Microplate Reader at the wavelength of 570 nm. The absorption of each well represented its cytoviability; the cytoviability of 0 concentrations were set as 1.0, and the other wells were normalized to 0 concentration wells. All processes were conducted in triplicate and the date are presented as means ± standard deviation (SD).

### 3.4. Hemolysis Assay

Red blood cells were drawn from mice [39,40]. Then, the cells were incubated with different concentrations (4, 2, and 1 μM, respectively, in 1× PBS) of Cy5-BF_3_(^19^F) in 1.5-mL tubes. The cells incubated with deionized water were used as the control. Four hours later, the tubes were centrifuged at 5000 rpm for 3 min, and then photos of the results were taken.

### 3.5. In Vitro Cell Imaging and FACS Quantification

A549 cells were seeded in 24-well plates and incubated with Cy5-BF_3_(^19^F) (2 μM) for 1 h in 5% CO_2_ at 37 °C in a humidified incubator. Hoechst 33258 at a final concentration of 1 μg/mL was added and incubated at 37 °C for another 1 min. Cells were washed with warm 1× PBS buffer (pH 7.4, 37 °C) three times. The cells were then imaged by fluorescence microscopy (EVOS FL Auto Cell Imaging System) at the Cy5 channel (Ex = 628/40; Em = 692/40) to collect the fluorescence of the conjugate, and at the DAPI channel (Ex = 357/44; Em = 447/60) to collect the fluorescence of Hoechst 33258.

Quantification of A549 cell labeling with FACS proceeded as follows. Suspended A549 cells were incubated with Cy5-BF_3_(^19^F) at different concentrations (0, 0.01, 0.1, and 1 μM, respectively) for 1 h in 5% CO_2_ at 37 °C in a humidified incubator. Cells were washed with warm 1× PBS buffer (pH 7.4, 37 °C) three times and then fluorescence was quantified with FACS (Beckman Coulter Gallios) (channel FL-6, 633 nm laser excitation and 660/20 nm band pass).

### 3.6. In Vivo PET Imaging and In Vitro Fluorescence Imaging

The A549 xenograft tumor model was established by the subcutaneous injection of 2 million cells. After 3 weeks, the tumor reached 8 mm in diameter and was used for in vivo PET imaging. 

~60 μCi of Cy5-BF_3_ (^18^F) solution (~200 μL in 1× PBS buffer, pH 7.4) was intravenously injected into A549 tumor-bearing mice (Bal b/c nude mice, female, 10 weeks old, Charles River Laboratories) which were generated by the subcutaneous injection of 2 × 10^6^ cells in ~100 μL mixture (1/1, *v*/*v*) of 1× PBS buffer (pH 7.4, 0 °C) and matrigel. Mice were imaged on an Inveon PET/CT (Siemens, Princeton, NJ, USA) 6 h post-injection. Mice were sacrificed and collected with organs for ex vivo fluorescence imaging with a Bruker Xtreme Imaging System (Ex = 640 nm, Em = 700 nm filter long pass). All procedures conducted in mice were approved by the Weill Cornell Medical Center Institutional Animal Care and Use Committee (#2014-0030, 14 February 2017), and were consistent with the recommendations of the American Veterinary Medical Association and the National Institutes of Health Guide for the Care and Use of Laboratory Animals.

## 4. Conclusions

We describe the synthesis of an ^18^F-labeled Cy5, whose radiosynthesis is achieved in a one-step, 1-h reaction. The labeled conjugate shows similar absorption and emission spectra to Cy5 dye, which has ideal absorption coefficients, quantum yields, and absorption/emission wavelengths that place it in an ideal NIR biological window for fluorescence bio-imaging. The in vitro demonstration of cell viability and cell staining indicates its safety and potential utility in clinical applications. An in vivo study shows some tumor PET signals and demonstrates the utility of Cy5-BF_3_ (^18^F) in tumor identification in non-invasive PET imaging. The ex vivo imaging of collected organs shows some fluorescent signals in a xenographed tumor, which demonstrates the potential of this molecule in fluorescence-guided surgery.

## Supplementary Materials

Supplementary materials can be found at www.mdpi.com/1422-0067/18/6/1214/s1.

## References

[1] Luo S., Zhang E., Su Y., Cheng T., Shi C. (2011). A review of NIR dyes in cancer targeting and imaging. Biomaterials.

[2] James N.S., Chen Y., Joshi P., Ohulchanskyy T.Y., Ethirajan M., Henary M., Strekowsk L., Pandey R.K. (2013). Evaluation of polymethine dyes as potential probes for near infrared fluorescence imaging of tumors: Part-1. Theranostics.

[3] Schaafsma B.E., Mieog J.S.D., Hutteman M., Van der Vorst J.R., Kuppen P.J., Löwik C.W., Frangioni J.V., van de Velde C.J., Vahrmeijer A.L. (2011). The clinical use of indocyanine green as a near-infrared fluorescent contrast agent for image-guided oncologic surgery. J. Surg. Oncol..

[4] Kitai T., Inomoto T., Miwa M., Shikayama T. (2005). Fluorescence navigation with indocyanine green for detecting sentinel lymph nodes in breast cancer. Breast Cancer.

[5] Stoffels I., Dissemond J., Pöppel T., Schadendorf D., Klode J. (2015). Intraoperative fluorescence imaging for sentinel lymph node detection: Prospective clinical trial to compare the usefulness of indocyanine green vs technetium Tc 99m for identification of sentinel lymph nodes. JAMA Surg..

[6] Verjans J.W., Osborn E.A., Ughi G.J., Press M.A.C., Hamidi E., Antoniadis A.P., Papafaklis M.I., Conrad M.F., Libby P., Stone P.H. (2016). Targeted near-infrared fluorescence imaging of atherosclerosis: Clinical and intracoronary evaluation of indocyanine green. JACC Cardiovasc. Imaging.

[7] Zhang C., Liu T., Su Y., Luo S., Zhu Y., Tan X., Fan S., Zhang L., Zhou Y., Cheng T. (2010). A near-infrared fluorescent heptamethine indocyanine dye with preferential tumor accumulation for in vivo imaging. Biomaterials.

[8] Yang X., Shi C., Tong R., Qian W., Zhau H.E., Wang R., Zhu G., Cheng J., Yang V.W., Cheng T. (2010). Near IR heptamethine cyanine dye-mediated cancer imaging. Clin. Cancer Res..

[9] Luo S., Tan X., Qi Q., Guo Q., Ran X., Zhang L., Zhang E., Liang Y., Weng L., Zheng H. (2013). A multifunctional heptamethine near-infrared dye for cancer theranosis. Biomaterials.

[10] Zhang E., Luo S., Tan X., Shi C. (2014). Mechanistic study of IR-780 dye as a potential tumor targeting and drug delivery agent. Biomaterials.

[11] Pansare V.J., Hejazi S., Faenza W.J., Prud’homme R.K. (2012). Review of long-wavelength optical and NIR imaging materials: Contrast agents, fluorophores, and multifunctional nano carriers. Chem. Mater..

[12] Wang L.V., Hu S. (2012). Photoacoustic tomography: In vivo imaging from organelles to organs. Science.

[13] Zhang C., Zhang F., Wang W., Liu J., Xu M., Wu D., Shuai X., Shen J., Cao Z. (2016). Chitosan coated gold nanorod chelating gadolinium for MRI-visible photothermal therapy of cancer. RSC Adv..

[14] Harrison V.S.R., Carney C.E., MacRenaris K.W., Waters E.A., Meade T.J. (2015). Multimeric near IR-MR contrast agent for multimodal in vivo imaging. J. Am. Chem. Soc..

[15] An F.-F., Chan M., Kommidi H., Ting R. (2016). Dual PET and near-infrared fluorescence imaging probes as tools for imaging in oncology. Am. J. Roentgenol..

[16] Ryu J.H., Koo H., Sun I.-C., Yuk S.H., Choi K., Kim K., Kwon I.C. (2012). Tumor-targeting multi-functional nanoparticles for theragnosis: New paradigm for cancer therapy. Adv. Drug Deliv. Rev..

[17] Yamaguchi H., Tsuchimochi M., Hayama K., Kawase T., Tsubokawa N. (2016). Dual-labeled near-infrared/^99m^Tc imaging probes using PAMAM-coated silica nanoparticles for the imaging of HER2-expressing cancer cells. Int. J. Mol. Sci..

[18] Kim J.S., Kim Y.-H., Kim J.H., Kang K.W., Tae E.L., Youn H., Kim D., Kim S.-K., Kwon J.-T., Cho M.-H. (2012). Development and in vivo imaging of a PET/MRI nanoprobe with enhanced NIR fluorescence by dye encapsulation. Nanomedicine.

[19] Xing Y., Zhao J., Conti P.S., Chen K. (2014). Radiolabeled nanoparticles for multimodality tumor imaging. Theranostics.

[20] Lee D.-E., Koo H., Sun I.-C., Ryu J.H., Kim K., Kwon I.C. (2012). Multifunctional nanoparticles for multimodal imaging and theragnosis. Chem. Soc. Rev..

[21] Serwotka-Suszczak A.M., Sochaj-Gregorczyk A.M., Pieczykolan J., Krowarsch D., Jelen F., Otlewski J. (2017). A conjugate based on anti-HER2 diaffibody and auristatin E targets HER2-positive cancer cells. Int. J. Mol. Sci..

[22] Xiao L., Zhang Y., Yue W., Xie X., Wang J.-P., Chordia M.D., Chung L.W.K., Pan D. (2013). Heptamethine cyanine based ^64^Cu-PET probe PC-1001 for cancer imaging: Synthesis and in vivo evaluation. Nucl. Med. Biol..

[23] Hu H., Huang P., Weiss O.J., Yan X., Yue X., Zhang M.G., Tang Y., Nie L., Ma Y., Niu G. (2014). PET and NIR optical imaging using self-illuminating ^64^Cu-doped chelator-free gold nanoclusters. Biomaterials.

[24] Chen F., Ellison P.A., Lewis C.M., Hong H., Zhang Y., Shi S., Hernandez R., Meyerand M.E., Barnhart T.E., Cai W. (2013). Chelator-free synthesis of a dual-modality PET/MRI agent. Angew. Chem. Int. Ed..

[25] Ting R., Adam M.J., Ruth T.J., Perrin D.M. (2005). Arylfluoroborates and alkylfluorosilicates as potential PET imaging agents:  High-yielding aqueous biomolecular ^18^F-labeling. J. Am. Chem. Soc..

[26] Ting R., Harwig C., auf dem Keller U., McCormick S., Austin P., Overall C.M., Adam M.J., Ruth T.J., Perrin D.M. (2008). Toward [^18^F]-labeled aryltrifluoroborate radiotracers: In vivo positron emission tomography imaging of stable aryltrifluoroborate clearance in mice. J. Am. Chem. Soc..

[27] Bernard-Gauthier V., Bailey J.J., Liu Z., Wängler B., Wängler C., Jurkschat K., Perrin D.M., Schirrmacher R. (2016). From unorthodox to established: The current status of ^18^F-trifluoroborate- and ^18^F-SiFA-based radiopharmaceuticals in PET nuclear imaging. Bioconjug. Chem..

[28] Perrin D.M. (2016). [^18^F]-organotrifluoroborates as radioprosthetic groups for PET imaging: From design principles to preclinical applications. Acc. Chem. Res..

[29] Lang K., Chin J.W. (2013). Fluorescent imaging: Shining a light into live cells. Nat. Chem..

[30] Weissleder R. (2001). A clearer vision for in vivo imaging. Nat. Biotechnol..

[31] Kaloyanova S., Zagranyarski Y., Ritz S., Hanulová M., Koynov K., Vonderheit A., Müllen K., Peneva K. (2016). Water-soluble NIR-absorbing rylene chromophores for selective staining of cellular organelles. J. Am. Chem. Soc..

[32] Khandelia R., Bhandari S., Pan U.N., Ghosh S.S., Chattopadhyay A. (2015). Gold nanocluster embedded albumin nanoparticles for two-photon imaging of cancer cells accompanying drug delivery. Small.

[33] Guo Z., Park S., Yoon J., Shin I. (2014). Recent progress in the development of near-infrared fluorescent probes for bioimaging applications. Chem. Soc. Rev..

[34] Mujumdar R.B., Ernst L.A., Mujumdar S.R., Lewis C.J., Waggoner A.S. (1993). Cyanine dye labeling reagents: Sulfoindocyanine succinimidyl esters. Bioconjug. Chem..

[35] Ting R., Aguilera T.A., Crisp J.L., Hall D.J., Eckelman W.C., Vera D.R., Tsien R.Y. (2010). Fast ^18^F labeling of a near-infrared fluorophore enables positron emission tomography and optical imaging of sentinel lymph nodes. Bioconjug. Chem..

[36] Rodriguez E.A., Wang Y., Crisp J.L., Vera D.R., Tsien R.Y., Ting R. (2016). New dioxaborolane chemistry enables [^18^F]-positron-emitting, fluorescent [^18^F]-multimodality biomolecule generation from the solid phase. Bioconjug. Chem..

[37] Auf dem Keller U., Bellac C.L., Li Y., Lou Y., Lange P.F., Ting R., Harwig C., Kappelhoff R., Dedhar S., Adam M.J. (2010). Novel matrix metalloproteinase inhibitor [^18^F] marimastat-aryltrifluoroborate as a probe for in vivo positron emission tomography imaging in cancer. Cancer Res..

[38] Li Y., Ting R., Harwig C.W., auf dem Keller U., Bellac C.L., Lange P.F., Inkster J.A.H., Schaffer P., Adam M.J., Ruth T.J. (2011). Towards kit-like ^18^F-labeling of marimastat, a noncovalent inhibitor drug for in vivo PET imaging cancer associated matrix metalloproteases. MedChemComm.

[39] Cao Z., Zhu W., Wang W., Zhang C., Xu M., Liu J., Feng S.-T., Jiang Q., Xie X. (2014). Stable cerasomes for simultaneous drug delivery and magnetic resonance imaging. Int. J. Nanomed..

[40] Zhang J., Li S., An F.-F., Liu J., Jin S., Zhang J.-C., Wang P.C., Zhang X., Lee C.-S., Liang X.-J. (2015). Self-carried curcumin nanoparticles for in vitro and in vivo cancer therapy with real-time monitoring of drug release. Nanoscale.

