# Correction: An, F., et al. A Conjugate of Pentamethine Cyanine and ^18^F as a Positron Emission Tomography/Near-Infrared Fluorescence Probe for Multimodality Tumor Imaging. *Int. J. Mol. Sci.* 2017, *18*, 1214

**DOI:** 10.3390/ijms20143584

**Published:** 2019-07-22

**Authors:** Fei-Fei An, Harikrishna Kommidi, Nandi Chen, Richard Ting

**Affiliations:** 1 Molecular Imaging Innovations Institute (MI3), Department of Radiology, Weill Cornell Medical College, 413 East 69th Street, New York, NY 10065, USA; 2 State Key Laboratory of Chemo/Biosensing and Chemometrics, College of Chemistry and Chemical Engineering, Key Laboratory for Bio-Nanotechnology and Molecular Engineering of Hunan Province, Hunan University, Changsha 410082, China

The authors wish to make the following corrections to this paper [1]: The authors regret the incorrect appearance of Figure 9. The figure caption and manuscript discussion of Figure 9 in the original publication are correct. Unfortunately, Figure 9 appears incorrectly. The semi-quantitative calculation of ex vivo cyanine fluorescence biodistribution (Figure 9b below) was missing. The mistake was generated during final editing following peer-review. The mistake did not affect the review process. Our correction does not change the conclusions of this manuscript.

The corrected Figure 9 is shown below (Figure 1).

The authors would like to apologize for any inconvenience caused to the readers by these changes.

## Figures and Tables

**Figure 1:**
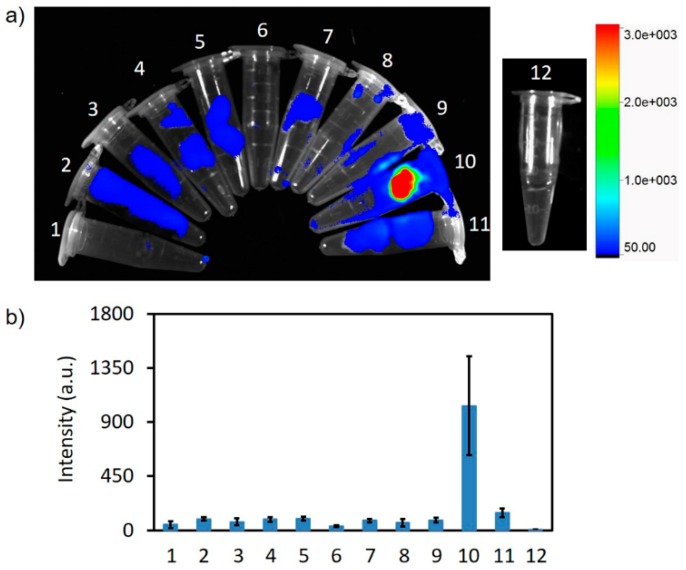
(**a**) Overlay of ex vivo fluorescence imaging and bright field image of the collected organs. (**b**) The semi-quantitative biodistribution by fluorescence imaging 6 h after intravenously injecting the Cy5-BF_3_ (^18^F). 1—Heart, 2—Liver, 3—Spleen, 4—Lung, 5—Kidney, 6—Stomach, 7—Intestine, 8—Bone, 9—Muscle, 10—Tumor, 11—Brain, and 12—1 × PBS.
