# Supplementary material for: A Conjugate of Pentamethine Cyanine and 18F as a Positron Emission Tomography/Near-Infrared Fluorescence Probe for Multimodality Tumor Imaging
Source: Int J Mol Sci. 2017 Jun 7;18(6):1214. doi: 10.3390/ijms18061214 (PMC5486037; doi:10.3390/ijms18061214)
Supplement: Supplementary file 1 [file ijms-18-01214-s001.pdf]

A conjugate of pentamethine cyanine and ^{18}F as a positron emission tomography/near-infrared fluorescence probe for multimodality tumor imaging

Fei-Fei An, Harikrishna Kommidi, Nandi Chen and Richard Ting

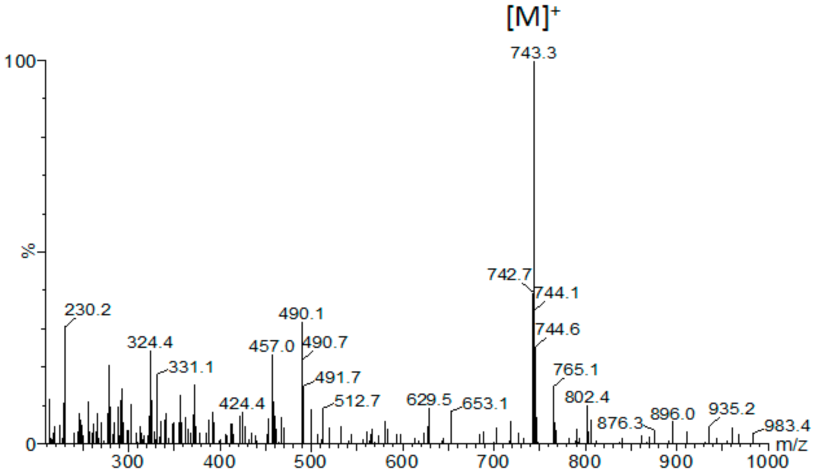

Figure S1. The mass spectrum of Cy5. Calculated for $[M]^+$ = $(C_{37}H_{47}N_2O_{10}S_2)^+$= 743.3 *m/z*, Observed $[M]^+$ = 743.3 *m/z*.

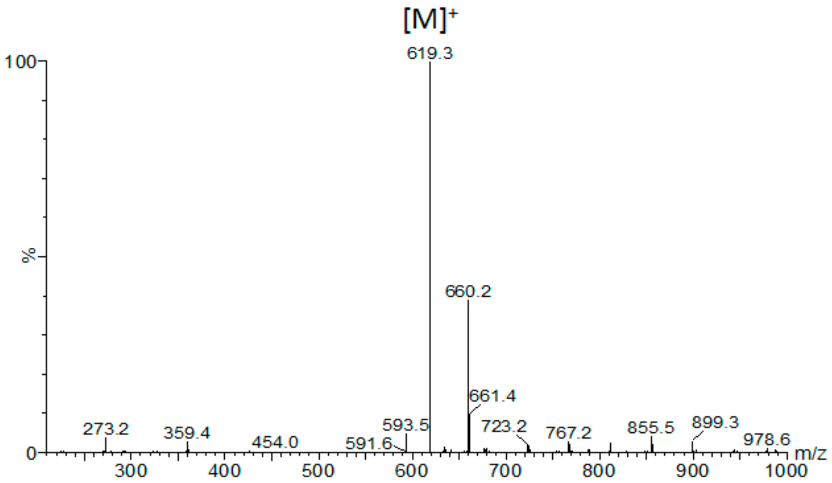

Figure S2. The mass spectrum of boronate. Calculated for $[M+H]^+$ $(C_{37}H_{31}BF_3N_2O_3)^+$= 619.2, Observed $[M]^+$ = 619.3 *m/z*.

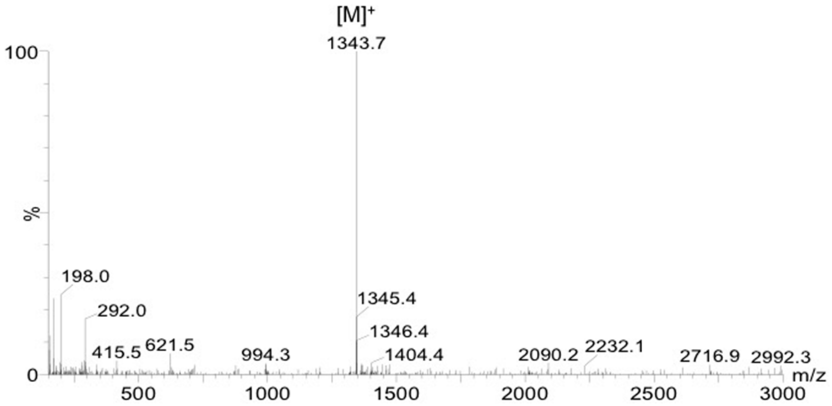

Figure S3. The mass spectrum of Cy5-B. Calculated for $[M]^+$= $(C_{74}H_{75}BF_3N_4O_{12}S_2)^+$ = 1343.5 *m/z*. Observed $[M]^+$ = 1343.7 *m/z*.

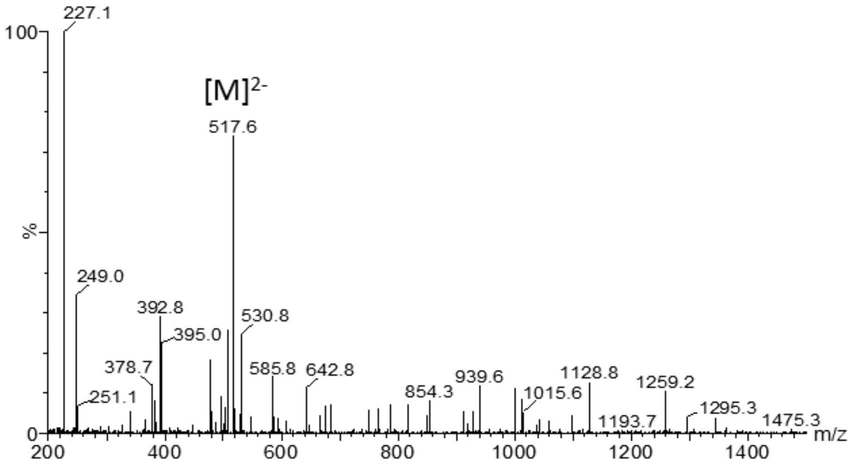

Figure S4. The mass spectrum of Cy5-BF3. Calculated for $[M]^-$= $(C_{48}H_{55}BF_6N_4O_{10}S_2)^-$ = 1034.3 *m/z*, $[M]^{2-}$ = 517.2 *m/z*, Observed $[M]^{2-}$ = 517.6 *m/z*.

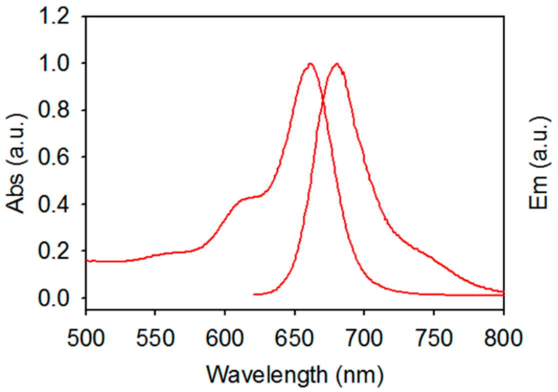

Figure S5. The absorption spectrum and emission spectrum of Cy5 in DMSO (Ex = 600 nm, Em = 680 nm).

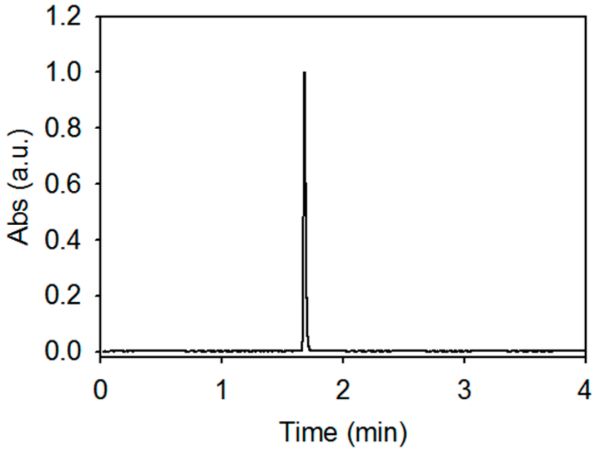

Figure S6. The HPLC profile of Cy5-B monitored at 650 nm wavelength (Reverse phase HPLC were performed on a Waters Acquity H class HPLC/ SQD2 mass spectrometer and a Phenomenex Kinetex 1.7 µm C18 100Å, 50 cm × 2.1 mm I.D. column (00B-4475-AN), with a 1.5 min, a10-90% H_2O:acetonitrile (ACN) (0.05% TFA) gradient and a flow rate of 0.6 mL/min.

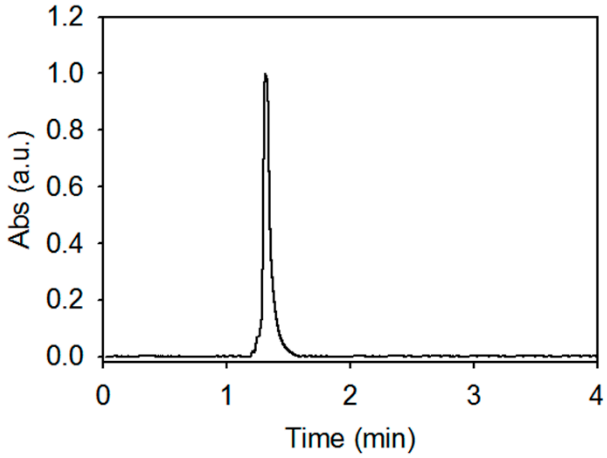

Figure S7. The HPLC profile of 19F-Cy5-BF_3 monitored at 650 nm wavelength (Reverse phase HPLC were performed on a Waters Acquity H class HPLC/ SQD2 mass spectrometer and a Phenomenex Kinetex 1.7µm C18 100Å, 50 cm x 2.1 mm I.D. column (00B-4475-AN), with a 1.5 min, a10-90% H_2O:acetonitrile (ACN) (0.05% TFA) gradient and a flow rate of 0.6 mL/min.

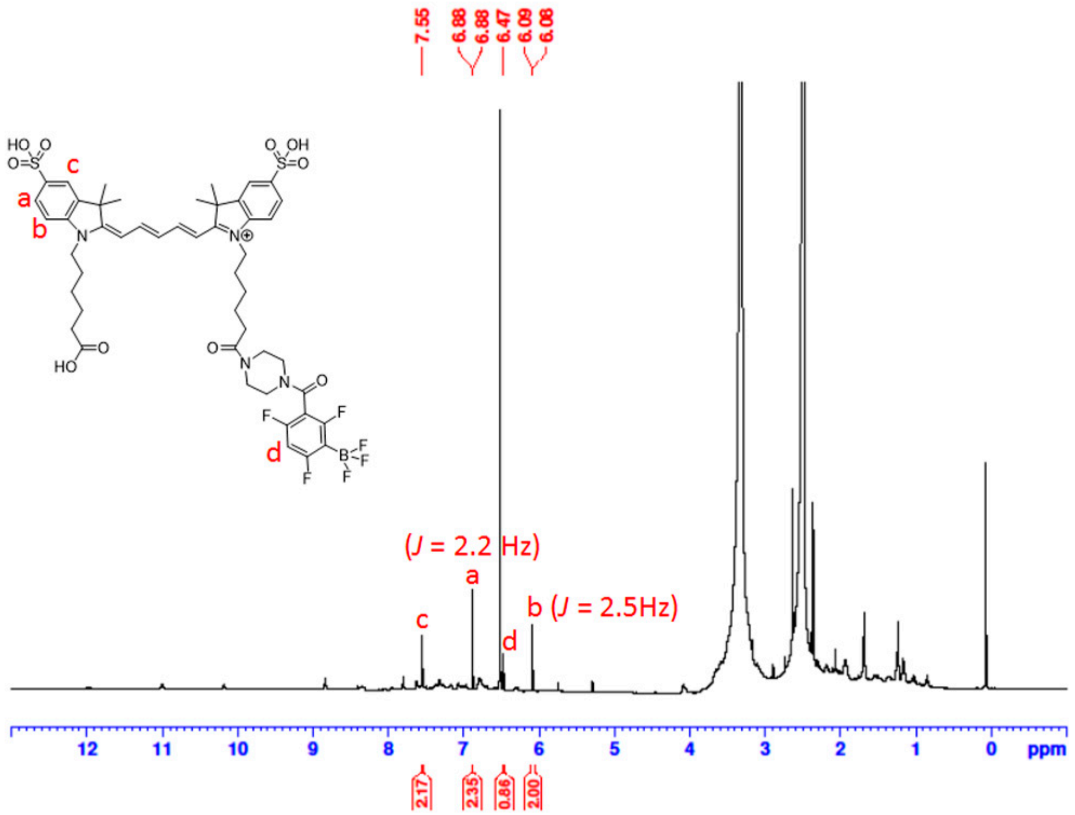

Figure S8. ^{1}H NMR (500 MHz, d6-DMSO, 21 °C, TMS) for Cy5-BF_3 (F-19).

Procedure for preparing of ^{18}F-ion water

One will need a glass v-vail (A Thermo Scientific Reacti-Vial #13223 is recommended), 40 psi nitrogen (from a low-pressure nitrogen cylinder), and a 1.2 M HF solution. We recommend working with low volumes of HF (< 200 μL).

In a typical labeling, a 700 μL volume of [^{18}F]-fluoride-ion-containing water produced from a 19.2 MeV bombardment of [^{18}O]-water is flushed from a cyclotron target into a Teflon septa-sealed 5.0mL glass v-vial (Thermo Scientific Reacti-Vial #13223). The vial is transferred to a 100 °C heat block and is flushed with 40 psi N_2 gas through an 18 G inlet. N_2 flow is vented through 16 G tubing and bubbled through a 1 M solution of NaOH to prevent radioactive volatilization. In 15 to 22 min, the solution of [^{18}F]-fluoride is concentrated to a ~20–30 μL volume. It is important not to dry the [^{18}F]-fluoride completely to obtain maximum [^{18}F]-fluoride recovery.

A 10 μL volume containing ~ 100 mCi of $^{18}F^-$ ion is added to 10 μL of a 1.2 M HF solution to give a 20 μL HF solution (0.6 M in water) containing 100 mCi of $^{18}F^-$ ion.

^{18}F-ion water Reaction with Cy5-B

The prepared ^{18}F-ion water solution is incubated with equivolume quantities of Cy5-B to generate the $^{18}F^-$ labeled fluorescent dye. A 1 h incubation at 50 °C is more than sufficient for complete Cy5-B conversion. This time is unoptimized. In the future, this synthesis can be made to proceed more rapidly using higher temperatures and/or a microwave reactor.
